# Effective control of acute myeloid leukaemia and acute lymphoblastic leukaemia progression by telomerase specific adoptive T-cell therapy

**DOI:** 10.18632/oncotarget.18115

**Published:** 2017-05-23

**Authors:** Sara Sandri, Francesco De Sanctis, Alessia Lamolinara, Federico Boschi, Ornella Poffe, Rosalinda Trovato, Alessandra Fiore, Sara Sartori, Andrea Sbarbati, Attilio Bondanza, Simone Cesaro, Mauro Krampera, Maria T. Scupoli, Michael I. Nishimura, Manuela Iezzi, Silvia Sartoris, Vincenzo Bronte, Stefano Ugel

**Affiliations:** ^1^ Department of Medicine, University of Verona, Section of Immunology, Verona, Italy; ^2^ Department of Medicine and Aging Science, Center of Excellence on Aging and Translational Medicine (CeSi-Met), G. D'Annunzio University, Chieti-Pescara, Italy; ^3^ Department of Computer Science, University of Verona, Verona, Italy; ^4^ Department of Neurological and Movement Sciences, University of Verona, Verona, Italy; ^5^ Innovative Immunotherapies Unit, Division of Immunology, Transplantation and Infectious Diseases, San Raffaele Hospital Scientific Institute, Vita-Salute San Raffaele University, Milano, Italy; ^6^ Department of Pediatric Haematology Oncology, University of Verona, Verona, Italy; ^7^ Department of Medicine, University of Verona, Section of Haematology, Verona, Italy; ^8^ University of Verona, Interdepartmental Laboratory for Medical Research (LURM), Verona, Italy; ^9^ Department of Surgery, Loyola University Medical Center, Maywood, IL, United States

**Keywords:** acute myeloid leukaemia (AML), B-cell acute lymphoblastic leukaemia (B-ALL), telomerase (TERT), TCR-redirected T-cells, adoptive cell therapy (ACT)

## Abstract

Telomerase (TERT) is a ribonucleoprotein enzyme that preserves the molecular organization at the ends of eukaryotic chromosomes. Since TERT deregulation is a common step in leukaemia, treatments targeting telomerase might be useful for the therapy of hematologic malignancies. Despite a large spectrum of potential drugs, their bench-to-bedside translation is quite limited, with only a therapeutic vaccine in the clinic and a telomerase inhibitor at late stage of preclinical validation. We recently demonstrated that the adoptive transfer of T cell transduced with an HLA-A2-restricted T-cell receptor (TCR), which recognize human TERT with high avidity, controls human B-cell chronic lymphocytic leukaemia (B-CLL) progression without severe side-effects in humanized mice. In the present report, we show the ability of our approach to limit the progression of more aggressive leukemic pathologies, such as acute myeloid leukaemia (AML) and B-cell acute lymphoblastic leukaemia (B-ALL). Together, our findings demonstrate that TERT-based adoptive cell therapy is a concrete platform of T cell-mediated immunotherapy for leukaemia treatment.

## INTRODUCTION

Acute myeloid leukaemia (AML) and B-cell acute lymphoblastic leukaemia (B-ALL) are still incurable diseases for a number of patients and a leading cause of death in children and adults, respectively [1–3]. Currently, the worldwide standard treatments, including induction chemotherapy followed by either consolidation chemotherapy, autologous or allogeneic stem cell transplantation, generated remission in a high percentage of treated patients: approximately, 90% of B-ALL patients diagnosed between 1 and 18 years of age are expected to be long-term and event-free survivors [4–6], and 70-80% of AML patients younger than 60 years can achieve complete remission [7, 8]. However, the outcome remains insufficient for patients who develop adverse disease-related events or treatment-related toxicity. In fact, the 40-50% of AML patients that undergo relapse showed a 5-year overall survival (O.S.) [9–11]. On the contrary, the 5-year O.S. of relapsed ALL patients was 10% in adults [12, 13] and 10-20% in children [9, 14]. In the last years, the immunotherapy of cancer has emerged as realistic treatment option for B-ALL and AML patients [15–17]. The minimal residual disease negative status of treated patients, which is around 79%, suggests that immunotherapy promotes long-lasting efficacy compared to standard procedures [18]. Also the use of Blinatumomab, bispecific T-cell engager (BiTE) directed towards CD19 and CD3 antigens, provided impressive clinical effects in B-ALL patients with either relapsed or refractory disease [19–21]. These exciting results in B-ALL have generated several adaptations to treat AML patients that are now under clinical evaluation. Since CD33 and CD123 markers are expressed on the majority of AML cells, they have been used to generate innovative tools such as CD33/CD3 (AMG330) [22, 23] or CD3/WT1-HLA-A^*^0201 [24] BiTEs or chimeric antigen receptors (CAR) targeting CD123 epitope [25–27]. BiTE demonstrated specific ability to contrast tumour progression in AML mouse models and a selective *in vitro* killing ability against human AML blasts, but currently no studies about toxicity has been reported yet. On the contrary, preclinical experiments using a selective hCD123 CAR able to eliminate human AML cells caused complete eradication of normal bone marrow (BM) cells in mice engrafted with human CD34^+^ stem cells [27]. These data also emphasize the deleterious effects on normal myeloid cells caused by the use of potent immune-based therapies specific for a widespread antigen and highlight the relevance of selecting the correct target for the development of anti-cancer immunotherapy.

Human Telomerase (TERT) has been identified as a common hallmark of cancer, since it plays a critical role in aberrant cell proliferation and immortalization in the majority of tumours [28]. Variable levels of telomerase have been detected in up to 85% of all AML [29–31] and, normally, relapsed AML patients showed highest telomerase activity [30]. Among all the subtypes of acute leukemia, B-ALL cells showed the greatest level of telomerase activity and the shortest telomeres, conditions generally associated with reduced response to therapy, faster leukemic progression and poor prognosis [32–34]. All these findings designate TERT as ideal tumour-associated antigen (TAA) that could be exploited to design a selective cancer immunotherapy for the treatment of leukemias [35]. Indeed, TERT generates immunogenic epitopes for both major histocompatibility complex (MHC) class I and II pathways, able to trigger an adaptive cytotoxic T lymphocytes (CTL) response against tumour cells [36, 37]. A spontaneous immune response against TERT was reported in different tumour settings and anti-TERT specific CD8^+^T cells were detected, with a higher frequency, in the blood of patients affected by chronic lymphocytic leukemia (B-CLL), as well as breast, lung and colorectal cancers, compared to healthy donors (HD) [38–42]. However, the endogenous anti-TERT T cells isolated from B-CLL normally display a very low affinity in their TCR, thus limiting their use in adoptive cell therapy (ACT) [38]. To overcome this limitation, we recently demonstrated the ability of hTERT_865-873_-specific, TCR-engineered T-cells both to efficiently recognize different solid human tumour cells and restrict human B-CLL tumour progression *in vivo* without inducing dramatic toxicity. In fact, the hTERT-specific ACT did not induce myeloid precursor depletion in tumour-bearing humanized mice, affecting only BM resident mature granulocytes and preserving the ability of hCD34^+^ cells to generate mature leukocytes [38]. We describe here the exploitation of the therapeutic efficacy of our anti-TERT-based ACT approach in more aggressive haematological cancer settings, such as AML and B-ALL, to validate its versatility as a widespread anti-tumour immunotherapy for leukemic diseases.

## RESULTS

### hTERT_865-873_-specific, TCR-engineered T-cells reduce AML progression *in vivo*

**Figure 1:**
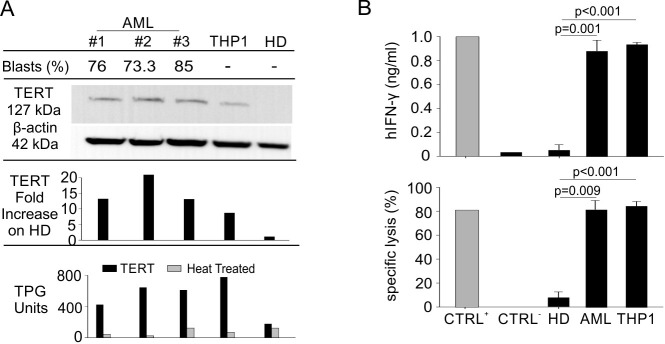
Engineered hTERT_865-873_-specific T-cells selectively recognize HLA-A2^+^ acute myeloid leukemia *in vitro* **A**. Percentage of leukemic blasts, telomerase expression (WB and lane quantification normalized on β-actin) and enzymatic activity levels in THP1 cell line, PBMCs from AML patients and age-matched HDs (representative cases out of 10 AML patients and 4 HDs are shown). **B**. AML leukemic cells were recognized *in vitro* by engineered hTERT_865-873_-specific T-cells upon 24-hour co-culture, as assayed both by hIFN-γ release assay (upper panel) and flow cytometry cytotoxicity assay (lower panel). Data are mean ± SD of three independent experiments: hTERT_865-873_ pulsed HLA-A2^+^ HD PBMCs (*n* = 3; CTRL+); hHCV_1406-1415_-pulsed HLA-A2^+^ HD PBMCs (*n* = 3; CTRL-); HLA-A2^+^ HD PBMCs (*n* = 4); HLA-A2^+^ PBMCs from AML patients (*n* = 10); THP1 cell line (*n* = 3). Statistical analysis was performed with ANOVA test.

In our recent work, we characterized the anti-cancer efficacy of hTERT_865-873_-specific, TCR-engineered T-cells transferred to human tumour-bearing immunocompromised (NOG) mice. In particular, we emphasized the ability of TERT-based ACT to promote a strong control of tumor growth in several cancer settings, while ignoring hematopoietic progenitors and activated T lymphocytes [38]. Conversely, hTERT_865-873_-specific, TCR-engineered T-cells specifically induced the contraction of the mature granulocytic cell subset resident in the BM of human immune reconstituted (HIR) mice [38]. Starting from these data, we explored the killing ability of our hTERT_865-873_-specific TCR-engineered T-cells on AML cells. We initially verified TERT expression and activity in both PBMCs isolated from HLA-A2^+^ AML patients or HD and THP1 human myeloid leukaemia-immortalized cells (Figure 1A). All leukemic cells showed a heightened TERT activity compared to normal leukocytes. Moreover, hTERT_865-873_-specific TCR-engineered T-lymphocytes were able to discriminate normal from tumour cells: in fact, *in vitro*, they efficiently recognized AML PBMCs and THP1 cells, but not HD PBMCs (Figure 1B). Indeed, hTERT_865-873_-specific TCR-engineered T-cells secreted interferon (IFN)-γ in response to patients’ samples, at similar levels to those reached using THP1 cells as target (Figure 1B, left panel). Cell recognition was confirmed by a cytofluorimetric cytotoxic assay in which leukemic cells were selectively lysed by hTERT_865-873_-specific TCR-engineered T cells (Figure 1B, right panel). The hTERT_865-873_ TCR-engineered T-cells are specific for the target antigen, since they recognized PBMCs pulsed with hTERT_865-873_ peptide but not PBMCs pulsed with control hHCV_1406-1415_ peptide (Figure 1B). These data highlight that AML blasts and tumour cells were able to process TERT protein to generate the HLA-A2 class I/hTERT_865-873_ peptide complex able to activate anti-TERT CTLs.

**Figure 2:**
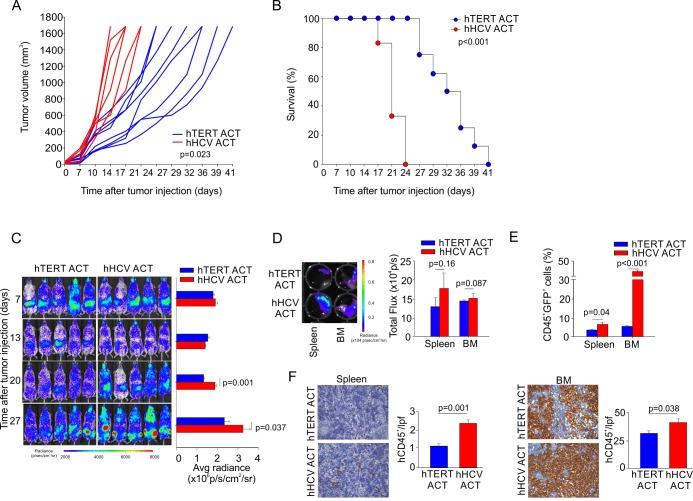
Engineered hTERT_865-873_-specific T-cells selectively recognize HLA-A2^+^ acute myeloid leukemia *in vivo* **A**. Assessment of hTERT-specific ACT capability to ameliorate mouse survival upon THP1-Luc subcutaneous challenge. Either hTERT_865-873_- or HCV_1406-1415_-TCR-engineered T cell were transferred twice, followed by the i.p. administration of IL-2 to test the ability to control tumor growth. **B**. Kaplan-Meier survival analysis (hTERT *n* = 8; hHCV *n* = 6) of a representative experiment. **C**. NOG mice were then intravenously injected with THP1-Luc cells and treated with three, weekly ACTs of hTERT_865-873_-specific or control HCV_1406-1415_-specific T-cells (representative data of 1 out of 2 independent experiments of total *n* = 12 mice per group). Tumor growth was weekly evaluated by bioluminescence imaging. **D**. At 27 days from tumor-challenge, leukemic cells spread to spleen and BM was tested by bioluminescence imaging. **E**. The percentage of infiltrating malignant hCD45^+^ cells was also evaluated by Flow cytometric analysis and **F**. by IHC (20X magnification). Statistical analysis was performed with Student's *t* test.

To test the therapeutic effect of hTERT_865-873_-specific TCR-engineered T-cells on controlling AML progression, we subcutaneously (s.c.) challenged immunodeficient NOG mice with THP1 cells. Our immunotherapeutic treatment based on anti-TERT CTLs infusion significantly controlled tumour growth inducing a survival benefit on treated mice compared to mice treated with hHCV_1406-1415_-specific TCR-engineered T-cells (Figure 2A-2B). Subsequently, we generated firefly luciferase-expressing THP1 cells (THP1-Luc) to track the *in vivo* spreading of AML cells after intravenous (i.v.) injection delivery, to mimic the disseminated disease in patients. THP1-Luc cells were recognized *in vitro* by the hTERT_865-873_-specific TCR-engineered T-lymphocytes at levels comparable with wild type (WT) THP1 cells (data not shown). Immunocompromised NOG mice were injected i.v. with 3×10^5^ THP1-Luc cells and treated with three weekly infusions of either hTERT_865-873_-specific TCR-engineered or hHCV_1406-1415_-specific TCR-engineered T-cells seven days after tumour challenge. Tumour progression was evaluated through bioluminescence imaging. Figure 2C demonstrates the ability of TERT-based ACT to significantly limit leukemic progression. We monitored tumour dissemination until day twenty-seven from tumour challenge when control group reached the endpoint threshold (3×10^6^ p/s/cm^2^/sr). Lymphopoietic organs (spleen and BM) isolated from control HCV-based ACT treated mice presented a more severe leukemic cell infiltration compared to TERT-based ACT treated mice (Figure 2D). Finally, to confirm the therapeutic effectiveness of TERT-based ACT, we analyzed THP1 accumulation, identified as human (h) CD45^+^ cells by flow cytometry analysis (Figure 2E). These data were confirmed also by immunohistochemistry (IHC), showing a significant contraction of leukemic cells in TERT-based ACT treated mice compared to controls (Figure 2F). All these findings suggest that TERT antigen can be pursued as a target for T-cell therapy of AML.

### hTERT_865-873_-specific TCR-engineered T-cells reduce ALL progression *in vivo*

**Figure 3:**
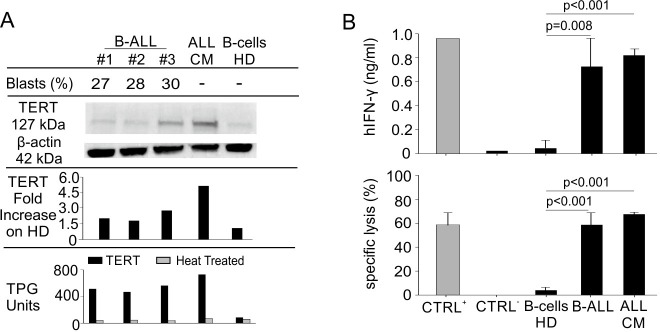
Engineered hTERT_865-873_-specific T-cells selectively recognize HLA-A2^+^ acute lymphoblastic leukemia *in vitro* **A**. Percentage of leukemic blasts, telomerase expression (WB and lane quantification normalized on β-actin) and enzymatic activity levels in ALL-CM cell line, B-cells isolated from PBMCs of B-ALL patients and age-matched HDs (shown some representative cases out of 10 B-ALL patients and 5 HDs). **B**. Leukemic cells isolated from B-ALL patients were recognized *in vitro* by engineered hTERT_865-873_-specific T-cells upon 24-hour co-culture, as assayed both by hIFN-γ release assay (upper panel) and flow cytometry cytotoxicity assay (lower panel). Data are mean ± SD of three independent experiments: hTERT_865-873_ pulsed HLA-A2^+^ HD B-cells (*n* = 3; CTRL+); hHCV_1406-1415_-pulsed HLA-A2^+^ HD B-cells (*n* = 3; CTRL-); HLA-A2^+^ HD B-cells (*n* = 5); HLA-A2^+^ B-cells from B-ALL patients (*n* = 10); ALL-CM cell line (*n* = 3). Statistical analysis was performed with ANOVA test.

**Figure 4:**
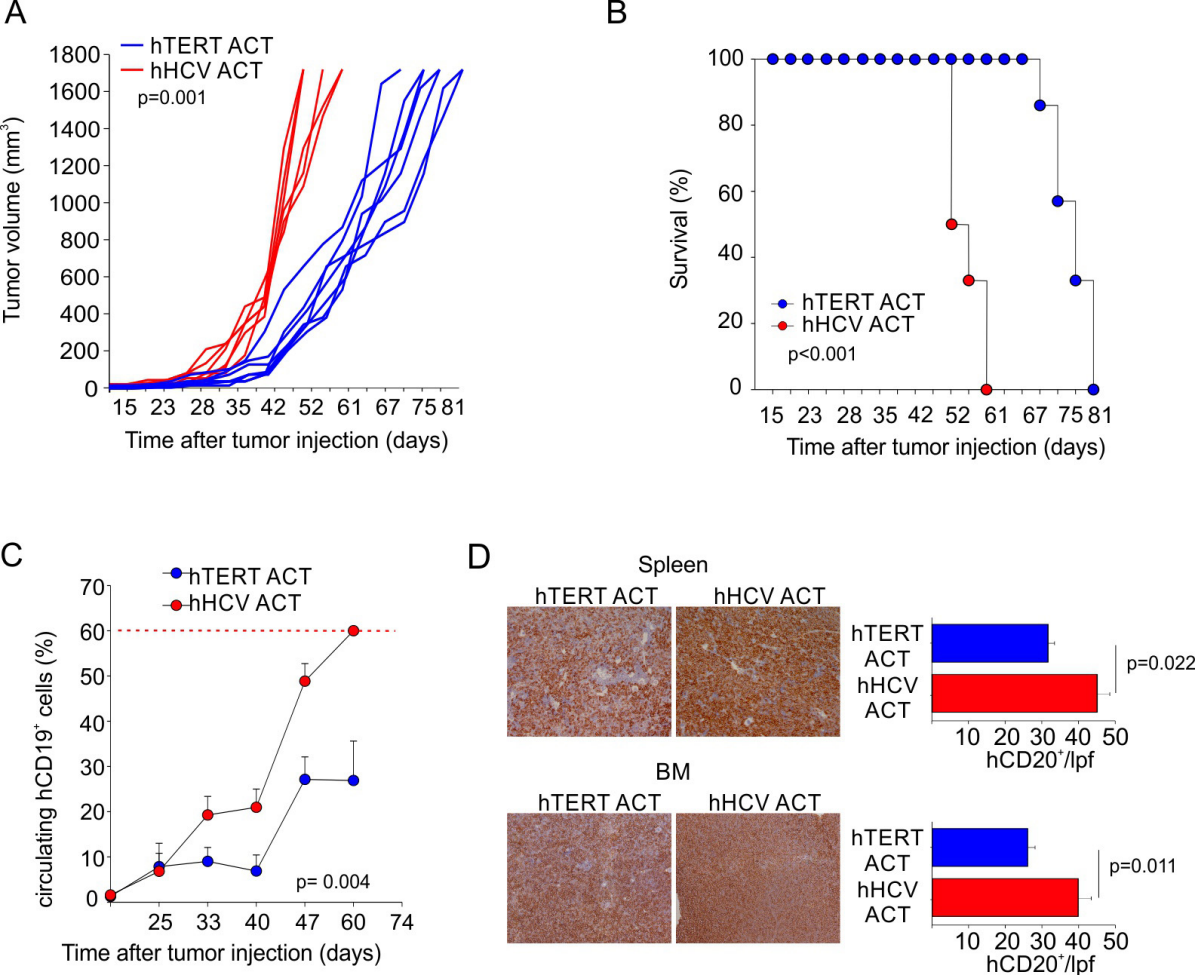
Engineered hTERT_865-873_-specific T-cells selectively recognize HLA-A2^+^ acute myeloid leukemia *in vivo* **A**. Assessment of hTERT-specific ACT capability to ameliorate mouse survival after ALL-CM subcutaneous challenge. Either hTERT_865-873_- or HCV_1406-1415_-TCR-engineered T cell were transferred twice, followed by the i.p. administration of IL-2 to test the ability to control tumor growth. **B**. Kaplan-Meier survival analysis (hTERT *n* = 7; hHCV *n* = 6) of a representative experiment. **C**. Assessment of hTERT-based ACT in controlling ALL-CM cells expansion upon intravenous injection. Mice were treated with three, weekly ACTs of hTERT_865-873_-specific or control HCV_1406-1415_-specific T-cells (cumulative graph of 2 independent experiments of total *n* = 12 mice per group). **D**. When control mice showed around 60% of circulating malignant cells (red dotted line), tumour cells dissemination into spleen and BM was tested by IHC (20X magnification). Data are mean ± SD of a representative experiment (*n* = 6 per group). Statistical analysis was performed with Student's *t* test.

**Figure 5:**
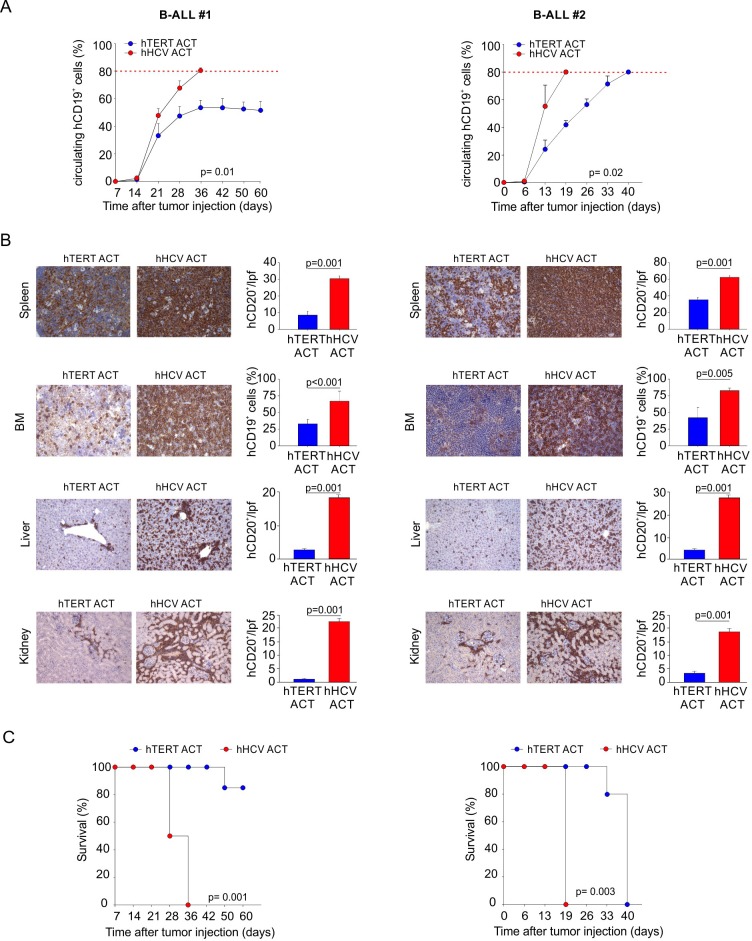
Engineered hTERT_865-873_-specific T-cells restrain human acute leukemia progression NOG mice were challenged with PBMCs isolated from two different HLA-A2^+^ B-ALL patients (B-ALL#1 and B-ALL#2). Mice were treated with 3 weekly ACTs of hTERT_865-873_- or hHCV_1406-1415_-specific T-cells, followed by IL-2 administration. **A**. Tumor progression was calculated evaluating circulating human B-cells. Mice where sacrificed when control mice showed around 80% of circulating malignant cells (red dotted line). **B**. IHC analysis of hCD20 expression in spleen, BM, liver and kidney (20X magnification). Only for BM (40X magnification), quantification was obtained by flow cytometric analysis. Data are mean ± SD. Statistical analysis was performed with Student's *t* test (*n* = 5 per group). **C**. Survival follow up of the remaining treated mice (B-ALL#1, hTERT *n* = 7, hHCV *n* = 5; B-ALL#2 hTERT *n* = 5, hHCV *n* = 4). Kaplan-Meier survival analysis: B-ALL#1, hTERT ACT *vs*. hHCV ACT: *p* = 0.001; B-ALL#2, hTERT ACT *vs*. hHCV ACT: *p* = 0.003.

**Figure 6:**
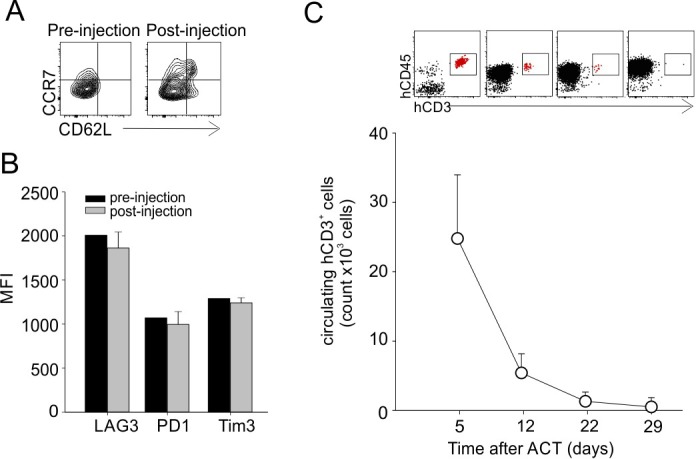
Phenotype and persistence of transferred T-cells *in vivo* **A**. hTERT_865-873_-specific T cells showed an effector memory phenotype (CD62L^−^CCR7^−^) before and 4 days after *in vivo* administration in leukemic mice. Data show representative contour plots. **B**. Analysis of engineered T cell inhibition markers (LAG3, PD1 and Tim3) pre-injection and 4 days post-injection in leukemic mice. **C**. Evaluation of circulating TERT-specific T cells at different time points after adoptive transfer in leukemic mice. Representative dot-plots are shown.

We previously demonstrated that hTERT_865-873_-specific TCR-engineered T-cells were able to significantly control B-CLL progression [38]. Once the efficacy of the adoptive transfer of TERT-specific T cells against human AML was confirmed, we investigated their antitumour activity towards human B-ALL. B-lymphocytes isolated from HLA-A2^+^ B-ALL patients and the HLA-A2^+^ ALL-CM immortalized cell line (both co-expressing hCD19 and hCD20 markers) were characterized by higher levels of telomerase expression and activity, when compared to B cells isolated from HD PBMCs (Figure 3A). Moreover, hTERT_865-873_-specific TCR-engineered T-cells efficiently recognized *in vitro* leukemic cells compared to normal B cells (Figure 3B). To confirm B-ALL targeting capability, NOG mice were s.c. challenged with ALL-CM cells and then treated with either hTERT_865-873_- or hHCV_1406-1415_ specific TCR-engineered T-cell infusion. The anti-TERT based immunotherapy significantly controlled tumour progression (Figure 4A) improving the survival of leukaemia-bearing mice (Figure 4B). To evaluate the therapeutic impact of TERT-targeting immunotherapy in a more relevant setting, NOG mice were i.v. injected with 2.5×10^6^ ALL-CM cells and tumour progression was weekly assessed by enumerating the frequency of circulating hCD19^+^ cells by flow cytometry, as previously described [43, 44]. We set up a threshold of ~60% of human B cells in the peripheral blood as disease endpoint. Three weekly administration of hTERT_865-873_-specific TCR-engineered T-cells significantly controlled ALL progression compared to control treatment (HCV-based ACT, Figure 4C). This therapeutic effect resulted in a significant reduction of tissue-infiltrating hCD20^+^ cells compared to controls (Figure 4D). For a translational perspective, we next evaluated the ability of TERT-based ACT to limit the *in vivo* progression of HLA-A2^+^ hCD19^+^ cells isolated from two different cryopreserved B-ALL patient's BM aspirates (referred as B-ALL#1 and B-ALL#2 respectively). We engrafted immunocompromised NOG mice with 1.5×10^6^ hCD19^+^ cells and then assessed the leukemic progression by quantifying the frequency of circulating hCD19^+^ lymphocytes. Mice were treated with three consecutive ACT, starting one week after tumour challenge. The TERT-based immunotherapeutic approach significantly controlled leukemic expansion compared to the control therapy (Figure 5A). To validate the therapeutic effect of TERT-based ACT, we sacrificed a cohort of leukemic mice when the control group showed around 60% of circulating hCD19^+^ lymphocytes. The IHC analysis of hCD20^+^ leukemic cells revealed a remarkable reduction in tumour accumulation in different tissues (spleen, BM, liver and kidney) in TERT-treated mice compared to control mice, therefore suggesting an effective control of leukemic spread (Figure 5B). Indeed, TERT-based ACT promoted a significant survival benefit (Figure 5C): in particular, in the case of mice engrafted with B-ALL#1 blasts, only the 16% of mice treated with hTERT-specific ACT developed, after 60 days from tumour challenge, an expansion of malignant human B cells that reached the threshold for sacrifice. The hTERT_865-873_-specific TCR-engineered T-cells (Figure 6A) exhibited a very modest *in vivo* persistence; in fact their presence in peripheral blood of treated mice decreased as soon as 7 days after injection (Figure 6B). These findings could in part explain the limited therapeutic impact on controlling tumour with more aggressive features in this experimental setting.

## DISCUSSION

The results of our study demonstrated that: a) AML and ALL cells not only overexpress TERT protein but also show an higher TERT activity compared to normal cells; b) AML and ALL cells process TERT protein, generate the immunodominant hTERT_865-873_ peptide and expose the immunogenic hTERT-HLA-A2 complex on the cell surface, promoting a specific T-cell recognition; c) hTERT_865-873_-specific TCR-engineered T-cells show specificity in recognizing both primary and immortalized AML and ALL cells but not target cells pulsed with unrelated antigen, normal PBMCs or B-cells; d) TERT-based ACT controls *in vivo* the systemic dissemination of AML and ALL cells; e) TERT-based ACT significantly prolongs the survival of ALL affected hosts. All these findings depict TERT-targeting immunotherapy as a promising strategy for treating leukemic patients.

Although the outstanding clinical success achieved by CAR-mediated immunotherapy in contrasting B-ALL progression has comprehensibly attracted the interest of scientific community for the development and improvement of this type of engineered T-cell therapy, the idea of redirecting T-lymphocytes with high affinity TCR selective for universal tumour-associated antigens, such as TERT, may open new avenues on the clinical procedure. Indeed, TERT is shared and actively expressed in about 85% of human tumours with various histological type [45]. Transformed tumour cells initially express TERT to acquire both an “immortalized” phenotype preventing cell apoptosis induced by the telomeres reduction and inducing self-renewal ability [46–48]. In the late tumorigenesis events, cancer cells develop mobilization and extravasation skills that promote the metastatic dissemination *via* TERT overexpression [49]. These findings demonstrate a key role for telomerase in different stages of tumour differentiation, therefore indicating a broad potential use of TERT-based ACT therapy. In agreement with this idea, our previous data showed both the ability of anti-TERT T lymphocytes to selectively destroy the pool of cancer stem cells (CSC), which usually survive conventional therapies favouring metastasis dissemination, and the therapeutic impact on controlling melanoma lung metastases [50]. We additionally proved the effectiveness of hTERT_865-873_-specific TCR-engineered T-cells ACT to control different human solid tumour growth [38]. Finally, we recently demonstrated the ability to further improve the therapeutic efficacy of TERT-based ACT by manipulating the cell composition in the tumour-microenvironment through the infusion of inducible nitric oxide synthase iNOS- and tumour necrosis factor (TNF) α-producing dendritic cells [51], as well as the use of either low dose of chemotherapy [52, 53] or drugs able to contain the generation of nitrogen reactive species [54], such as peroxynitrites [55], within the tumour microenvironment to defeat the immunosuppressive cancer barriers, favouring the anti-tumour function and persistence of the infused T cells.

The limited therapeutic impact of our immunotherapy is probably due to the low *in vivo* persistence of hTERT_865-873_-specific TCR-engineered T-cells rather than their exhaustion. In fact, before their *in vivo* administration, TCR-transduced T-cells had an effector/memory phenotype (CD45RA^−^CD62L^−^CCR7^−^ cells) characterized by the low-expression of exhaustion markers (LAG3/PD-1/Tim-3). Other immune-evasion mechanisms, such as antigen loss or antigen presentation deficiency, seem not to play a critical role in our experimental setting since residual leukemia cells in TERT-treated mice maintained telomerase expression and HLA-A2 molecules (data not shown). As recently reported, the identification and characterization of T memory stem cells (T_SCM_) generated an increased interest for their therapeutic use for cancer treatment. T_SCM_ cells, that are the earliest developmental stage of memory T cells, exhibit stem cell-like properties and display a gene profile between naïve and central memory T cells [56]. Moreover, T_SCM_ cells show in preclinical models an intense self-renewing capacity following antigen loss, a long lifespan since they are refractory to apoptosis and a robust proliferative potential [57] but overall, as described by Biasco and colleagues, transferred genetically modified T_SCM_ cells in human showed an extended longevity persisting in the host for up to 12 years post infusion [58]. T_SCM_ cells *in vivo* persistence not required cytokine supply [58]; therefore, ACT based on engineered T_SCM_ cells might completely eliminate all the adverse effects produced by the administration of high dose of IL-2 [59]. We plan to generate, in the next future, hTERT_865-873_-specific TCR-engineered T_SCM_-cells, taking advantage of recent protocols for T_SCM_ cells *in vitro* isolation and expansion, to provide their increased benefit on tumor control [56, 60], in association with checkpoint inhibitors, in order to design more appealing immunotherapeutic strategies. Obviously, a second limitation to envision the real impact of TERT-based therapy is linked to the selected xenograft mouse models, which do not recapitulate the integrated biology of an intact human immune system and the cross-talk between human transferred T cells with human stromal and endothelial cells, as well as with other human leukocyte subsets such as monocytes, granulocytes, macrophages, NK cells and T regulatory cells [61, 62]. A partial mitigation of this limitations could come from the creation of novel leukemia-affected, humanized mice, which permit *in vivo* analysis of anti-leukemic cell-based therapy in the presence of a human immune system [63].

The most serious concern about the impact of TERT-based ACT in clinic is related to its safety. In fact, even if tumour tissues showed a higher TERT expression and telomere length compared to normal tissues, it is impossible to predict whether and to what extent normal cells would be recognized by TERT-specific T cells. Prudence is needed when designing a selective TERT-targeting immunotherapy, as pointed out by our previous data about the treatment of prostate tumor-bearing mice with three cycles of ACT with mouse TERT-specific CD8^+^ T cells that caused a transient B-cell lymphopenia. This toxicity might be related to the schedule and type of immunotherapeutic approach. In fact, the side effects were observed when repeated infusions of TERT-specific CTLs were associated with the injection of an adenovirus encoding mouse TERT protein and the administration of high dose of IL-2, whereas DNA vaccination did not induce any detectable adverse event [50, 64]. However, to address issues about safety, it will be mandatory to assess the *in vitro* activation of hTERT_865-873_-specific TCR-engineered T-cells in contact with vital tissue slides of specimens with different histology. Moreover, to limit an uncontrolled activation of our transduced T cell that may produce severe immunopathologies, we plan to incorporate a suicide gene in the retroviral vector encoding TERT-specific TCR sequences. Indeed the use of an inducible caspase 9 (iCas9) “safety switch” has already produced excellent results on controlling graft-*versus*-host disease (GVHD) development in patients of haploidentical stem-cell transplants [65].

Collectively, our data proved and reinforced the potential clinical translation of TERT-based immunotherapy on treating different leukemic pathologies.

## MATERIALS AND METHODS

### Mice

NOG mice (NOD.Cg-*Prkdc*^scid^*Il2rg*^tm1Sug^/JicTac) were purchased from Taconic. All animal experiments were approved by Verona University Ethical Committee (http://www.medicina.univr.it/fol/main?ent=bibliocr&id=85) and authorized by Ministerial Decree (16/2014-B). B-ALL animal experiments were in accordance with the Amsterdam Protocol on animal protection and welfare and conducted according to the guidelines of Federation of European Laboratory Animal Science Associations (FELASA).

### Primary and immortalized cell lines

PBMCs isolated from AML patients and HDs and BM (BM) cells from B-ALL were collected at the Hematology Unit, Azienda Ospedaliera Universitaria Integrata (AOUI) in Verona (Italy). All participating people provided written informed consent for the collection and use of their samples for research purposes, in compliance with the Declaration of Helsinki. The study was approved by the local Ethics Committee (Comitato Etico per la Sperimentazione, AOUI of Verona, n. 1496). THP1 acute monocytic leukemia cell line were obtained from American Type Culture Collection (ATCC), and were maintained in RPMI 1640 medium (Lonza). ALL-CM acute lymphocytic leukemia cell line were provided by Dr. A. Bondanza under restricted condition with Leiden University Medical Center (Leiden, The Neatherland) [43] and cultured in X-VIVO medium (Lonza) containing 2% human serum (Gibco). Both media were also supplemented with 2 mM L-Glutamine, 10 mM HEPES, 100 U/ml Penicillin, and 100 U/ml Streptomycin (all reagents were purchased from Lonza). All cell lines were maintained at 37°C in a 5% CO_2_ atmosphere.

### Generation of hTERT_865-873_ - and hHCV_1406-1415_- specific T cells

hTERT_865-873_ -specific TCR sequences were isolated as previously described in [38]. As control, T cells engineered with a transgenic TCR specific for epitope hHCV_1406-1415_ (KLVALGINAV) were used. hTERT_865-873_ - and hHCV_1406-1415_- specific T cells were obtained by transduction of OKT-3-activated PBMCs with the viral supernatant of hTERT_865-873_/PG13 or HCV_1406-1415_/PG13 cell lines in the presence of hIL-15 (hIL-15, 100 ug/ml, Miltenyi) and rIL-2 (rIL-2, 300 IU/ml; Peprotech). Selected T cells were then expanded in AIM-V medium (Gibco) supplemented with 5% human serum (Gibco) with OKT-3 (30 ng/ml; eBioscience), recombinant IL-2 and human IL-15. The percentage of CD4^+^ and CD8^+^ T cells in the culture was always tested before *in vitro* or *in vivo* studies. In general, CD8^+^ T lymphocytes represent about 70-80% of the total T cells and numbers for *in vivo* treatments were adjusted in order to inject 2.5×10^6^ CD8^+^ T cells.

### Telomerase expression and activity

Telomerase expression was assessed by western blotting as previously described [38]. Telomerase activity was measured using TRAP assay (TRAPeze^®^ XL telomerase detection kit; Millipore), following manufacturer's instructions.

### Immunophenotyping

Single-cell suspensions were labelled with either fluorochrome-conjugated Ab anti-mouse CD45.1 (A20) or Ab anti-human CD3 (OKT-3), CD19 (HIB19), CD45 (2D1), HLA-A2 (BB7.2), purchased from either Biolegend or eBiosciences and the relative isotype controls purchased from the same companies. B-ALL samples were acquired with a FACSCanto II (BD) and analyzed with FlowJo software (Treestar Inc.).

### Isolation of hCD19^+^ cells from BM of ALL patients

Crio-preserved BM aspirates from ALL patients were thawed and the total cells were sorted with hCD19-Microbeads (Miltenyi) according with the standard procedures. Purified cells were analyzed by flow citometry and they reached the ~95% of CD19^+^ cells.

### Functional assays

IFN-γ secretion was quantified by a sandwich enzyme-linked immunosorbent assay (ELISA; eBioscience) according to manufacturer's instructions. Briefly, 3×10^5^ engineered CTLs were co-cultured at 3:1 ratio in presence of with human malignant cells (PBMCs isolated from AML patients, purified hCD19^+^ cells isolated from BM of B-ALL patients, THP1 or ALL-CM cell lines) or control cell targets (PBMCs or purified hCD19^+^ cells isolated from HDs). Negative and positive controls were represented by HD PBMCs pulsed with either hHCV_1406-1415_ or hTERT_865-873_ peptides, respectively. After 24 hours (h) of co-culture, the supernatants were harvested to measure the released IFN-γ. Moreover, functional response was also evaluated by flow cytometric cytotoxic activity assay as previously described [38]. In brief, target cells were firstly stained with 0.2 μM or 2 μM CFSE or CellTrace (both from Invitrogen) allowing the evaluation of four different cell targets simultaneously. Mixed targets were incubated at 2:1 ratio with effectors lymphocytes for 18 h and then analyzed by flow cytometry. The formula: 1- [(% pre negative control / % pre sample) /(% post negative control / % post sample)]x100; was used to determine specific lysis.

### Systemic treatment of subcutaneous tumours

For *in vivo* testing of the therapeutic activity of hTERT_865-873_-specific T cells, 5×10^6^ THP-1 or 5×10^6^ ALL-CM (in Matrigel - Corning, USA) cells were inoculated s.c. in the left flank of NOG mice. Tumour volume was calculated according to the following equation: V (mm3) = (d2 × D)/2, where d (mm) and D (mm) are the smallest and largest perpendicular tumour diameters, respectively, as assessed by caliper measurement. Treatments started when tumour volume reached 100 mm^3^. hTERT_865-873_- or control HCV_1406-1415_-specific T cells were injected i.v. in the tail vein once a week, for 2 consecutive weeks. Every adoptive transfer was followed by the administration of recombinant human IL-2 (30,000 IU/mouse) intraperitoneally every 12h, for a total of 6 doses.

### ACT on human B-ALL setting

Eight-week-old NOG mice were intravenously (i.v.) injected with ALL-CM cells (2.5×10^6^) or with hCD19^+^ cells isolated from a HLA-A2^+^ B-ALL patient (1.5×10^6^). Mice were then treated with three, weekly ACT of hTERT_865-873_-specific or control HCV_1406-1415_-specific T-cells starting one week after tumour challenge, followed by IL-2 administration, as previously indicated. Leukemic spread was monitored as percentage of circulating hCD19^+^ cells in peripheral blood and we set up a survival threshold of hCD19^+^ circulating leukemic cells in the amount of either 60% in the case of ALL-CM tumour setting or 80% in the case of patient derived ALL tumour setting.

### ACT on human AML setting

NOG mice (8-10 weeks of age) were firstly injected i.v. with 3×10^5^ THP1-LUC cells obtained by infection of lentivirus built up with the pLENTI.EGFP.Luc vector, kindly provided by Dr. D. Melisi (University of Verona). THP1-Luc cells were sorted to obtain a 95% of EGFP^+^ cell population that was used for the study. A total of 3 infusions of 2.5×10^6^ hTERT_865-873_- or hHCV_1406-1415_-specific CTLs were retro-orbitally injected every 7 days, starting 1 week after tumour injection, followed by IL-2 administration, as previously indicated. Leukaemia burden was monitored by BLI (photons/second/cm^2^/sr) using the IVIS Spectrum Imaging System (PerkinElmer). Images were acquired prior to treatment and then weekly, 7 days after the last treatment. Animals were anesthetized with isoflurane/oxygen. For each animal, dorsal and ventral images were obtained pre and 10 minutes after i.p. administration with 150 mg/kg of D-luciferin (PerkinElmer) in PBS in order to discriminate specific light signal from basal emission. Five mice were imaged simultaneously with the subsequent parameters: exposure time = 5 minutes, field of view = 19×19 cm, binning B = 8 and f/stop = 1. Images were quantified tracing the region of interest (ROI) on the entire animal body. Living Image Software 4.4 (PerkinElmer) was used to acquire and quantify the bioluminescence.

### Histological and immunohistochemical analysis

Spleen and BM samples were fixed in 10% neutral buffered formalin; after fixation, BM samples were decalcified with 10% EDTA (pH 7.4) for 3 weeks and embedded into paraffin; 5 μm slides were cut and stained with Hematoxylin (BioOptica) and Eosin (BioOptica) for histological examination. For immunohistochemistry, sections were deparaffinized, serially rehydrated and after the appropriate antigen retrieval procedure, incubated with the following primary antibodies: monoclonal Mouse Anti-Human CD45 (M0701, Dako Corporation) or monoclonal Mouse Anti-Human CD20 (M0755, Dako Corporation), followed by the secondary antibody Dako EnVision System-HRP Labelled Polymer Anti-mouse (K4001, Dako Corporation). After chromogen incubation, slides were counterstained in Hematoxylin and images were acquired by Leica DMRD optical microscope (Leica). The absence of cross-reactions between human and mouse antigens was verified testing anti-human antibodies on a normal mouse spleen. The percentage of positive cells was evaluated on the digital images of 5-6 samples per group (6-10 × 200 microscopic fields per sample) by 2 pathologists, independently and in a blind fashion.

### Statistical analysis

Data were indicated as the mean ± SD. Student's t test was used to determine statistically significant differences between two treatment groups, while ANOVA test was used in case of multiple comparisons. Growth curves were analyzed with Repeated Measures (RM) ANOVA. Survival analysis was performed using the Kaplan-Meier survival analysis (Log-Rank) method. *P* values less than 0.05 were considered statistically significant.
